# Safety of a New Chewable Formulation of Mebendazole for Preventive Chemotherapy Interventions to Treat Young Children in Countries with Moderate-to-High Prevalence of Soil Transmitted Helminth Infections

**DOI:** 10.1155/2012/590463

**Published:** 2012-12-24

**Authors:** Andrew J. Friedman, Said M. Ali, Marco Albonico

**Affiliations:** ^1^Janssen Research & Development, LLC, Titusville, New Jersey, NJ 08560, USA; ^2^Public Health Laboratory, Ivo de Carneri, P.O. Box 122, Pemba Island, Zanzibar, Tanzania; ^3^Ivo de Carneri Foundation, Viale Monza 44, 20127 Milan, Italy

## Abstract

The primary objective was to evaluate the safety and tolerability of the new chewable formulation of mebendazole to treat soil-transmitted helminth (STH) infections in children ≤10 years old with the goal of using this formulation in preventive chemotherapy programs and expand treatment to young children who are unable to swallow solid tablets. In this open-label, single-arm, phase 3 study conducted at Pemba Island, Zanzibar, Tanzania, children aged 2 to 10 years (median age: 4 years) were administered a single dose of the mebendazole 500 mg chewable tablet. Safety was assessed 30 minutes after dose and 3 days later. Of the 390 (98%) children who completed the study, 195 (55%) had ≥1 STH infection and 157 (45%) had no infection at baseline. The most common STH infections were *Trichuris trichiura* (51%), hookworm (16%), and *Ascaris lumbricoides* (7%). Treatment-emergent adverse events (TEAEs) were experienced by 11% of children. There was no difference in the percentage of children experiencing TEAEs between the age strata of 2–5 years and 6–10 years. Diarrhea was reported only in children aged 2–5 years. No correlation was observed between the type or percentage of AEs and presence or severity of infection. A single dose of mebendazole 500 mg chewable tablet was safe and well tolerated in children aged 2 to 10 years.

## 1. Introduction

Soil-transmitted helminth (STH) infections (i.e., *Ascaris lumbricoides*, *Trichuris trichiura,* and hookworm (*Necator americanus* and *Ancylostoma duodenale*)), a subgroup of neglected tropical diseases (NTDs), affect approximately 1.4 billion people worldwide, primarily children in the tropical and subtropical areas [1]. Infections by these intestinal parasites frequently result in diarrhea, malnutrition, anemia, stunted growth, impaired cognitive development, reduced school attendance and performance in children, and, hence, decreased productivity as adults [2, 3]. 

According to recent World Health Organization (WHO) estimates, about 883 million children worldwide require treatment for these diseases, of whom 300 million have severe morbidity [4]. In an attempt to control these infections, WHO launched a preventive chemotherapy (PC) strategy based on mass drug administration programs (MDAs) for deworming vulnerable high-risk groups such as pre-school children, school-aged children, and women of child-bearing age, through several public-private partnerships with pharmaceutical companies [5]. One such initiative is the Children Without Worms (CWW) program, established in 2005 as a joint venture of the Task Force for Child Survival and Development and Johnson & Johnson, which has used a 500 mg mebendazole solid oral tablet in its MDAs since 2007 [6, 7] and now has pledged the donation of 200 million tablets of mebendazole every year to scale up STH control. 

Mebendazole 500 mg tablet single-dose administration is approved for the treatment of STH infections in pediatric and adult populations [8] and has been marketed for more than 40 years. Although mebendazole is effective against STH infections, reinfection is almost certain to occur in endemic areas due to contaminated water and poor sanitation. For this reason, the goal of STH PC programs is not the cure of the disease but rather the control of disease severity by reducing and keeping the worm load of infected individuals below the threshold that is associated with morbidity through regular deworming treatments [8]. In addition, decreased worm loads reduce disease transmission to family and community members.

A single-dose regimen makes this product ideally suited for PC interventions. The only recognized limitation of this medication provided as a solid tablet is that younger children, particularly those <5 years of age, may not be able to swallow the tablet. The WHO recommends chewable tablets for children <5 years old; for those under 3 years of age, that chewable tablets are to be crushed and administered with a small amount of water to reduce the choking hazard [8]. A new mebendazole 500 mg chewable tablet was hence developed to expand the treatment of children down to age 1 year in PC campaigns and improve the ease of use in young children who may not be able or willing to swallow the tablet. 

The clinical pharmacokinetics of the 500 mg chewable mebendazole tablet was previously studied in 18 healthy adult volunteers. The plasma concentrations (maximum concentration (*C*_max_) and area under the curve (AUC) of the mebendazole 500 mg chewable tablet were found to be 63% and 100% higher, respectively, compared with the currently marketed mebendazole 500 mg solid tablet (data on file). However, due to the drug's inherent poor bioavailability (<10%), the vast majority of chewable mebendazole still remains in the intestinal lumen where it exerts its therapeutic effect [9, 10]. Since mebendazole is poorly absorbed, increases in peak serum drug concentrations and total drug exposure of 63% to 100% observed with the new mebendazole 500 mg chewable formulation are not likely to substantially change the intraluminal gastrointestinal concentration of mebendazole, and, thus, efficacy with the chewable formulation is expected to be similar to that of the solid formulation tablet. However, because the chewable tablet is associated with a higher systemic exposure, the current study was undertaken to evaluate the safety and tolerability of this chewable tablet in children aged 2 to 10 years of age in a moderate-to-high prevalence area for STH infections, before it could be considered for PC interventions MDA programs.

## 2. Materials and Methods

### 2.1. Study Population

The study included children aged 2 to 10 years living in a high prevalence area (Pemba Island, Tanzania) where parasitic infections are endemic. Based on the medical history, physical examination, and vital signs, all enrolled children were healthy; all were able to chew the mebendazole chewable tablet. All girls enrolled in the study were premenarchal. The exclusion criteria included history of clinically significant liver or renal insufficiency; suspected massive intestinal parasitic infection; hypersensitivity to mebendazole or any inert ingredients in the chewable formulation or other medications in the benzimidazole class; treatment with therapies cimetidine and antiepileptics, such as phenytoin, which could affect the bioavailability of mebendazole; history of consumption of any medication containing mebendazole or any other treatment for STH infection within 30 days before entering into the study.

The study protocol was approved by the Zanzibar Medical Research Council ethics committee and the study was conducted in accordance with the ethical principles originating in the Declaration of Helsinki and in accordance with the International Conference on Harmonization Good Clinical Practice guidelines, applicable regulatory requirements, and in compliance with the respective protocols. The child's parents or their legally accepted representatives provided written informed consent before participation in the study. 

### 2.2. Study Design

This was an open-label, single-center, single-dose, safety study conducted at Pemba Island in Zanzibar, Tanzania consisting of a screening period and an open-label treatment phase of 3 days. On day 1 of the open-label phase, the eligible children were administered a mebendazole 500 mg chewable tablet by the study staff who ensured that the tablet was properly chewed and swallowed.

The degree of infection was determined by egg counts in baseline stool sample, as assessed by the Kato-Katz methodology [11]. The children were considered to have no infection if there were no eggs seen on evaluation; light infection if the number of eggs per gram of stool sample was 1–4,999 for *A. lumbricoides*, 1–999 for *T. trichiura*, 1–1,999 for hookworm; moderate infection if the eggs per gram of stool sample was 5,000–49,999 for *A. lumbricoides*, 1,000–9,999 for *T. trichiura*, 2,000–3,999 for hookworms, according to the WHO classification [12]. For children infected with multiple parasites, the classification was done according to the most severe infection. 

### 2.3. Safety Assessments

The safety analysis set included all children who received the study drug. Safety was evaluated in two age strata (children aged 2–5 years old and children aged 6–10 years old) by examining the incidence, severity, relationship to study drug and type of adverse events, and change from baseline to the end of study/early withdrawal visit in physical examination and vital sign measurements. Treatment-emergent adverse events (TEAEs) were recorded at 30 minutes after dose and 3 days later. TEAEs were assessed by direct observation of the children, reported by the parent or guardian, or both. Stool samples collected at baseline (before dose) were utilized as a means to correlate the degree of infection in children with the frequency and severity of TEAEs. Safety data were analyzed and stratified by the baseline egg counts. Medications that could affect bioavailability of mebendazole were not allowed. These included, but were not limited to cimetidine, antiepileptics such as phenytoin, ethotoin, and carbamazepine. 

### 2.4. Statistical Analysis

A total of 375 children were planned to be enrolled into the study assuming a 20 percent drop-out rate, to ensure that approximately 300 children would complete the study. This would give a 90 percent probability to detect adverse events that occurred with incidence rates of one percent. 

Frequency and severity of adverse events were correlated with the intensity of helminth infection based on the baseline stool sample quantification evaluations. For each adverse event, the percentage of children who experienced at least 1 occurrence of the given event was summarized.

## 3. Results

### 3.1. Patient Disposition and Baseline Characteristics

This study was conducted from 24 February 2010 to 12 March 2010. A total of 396 children were enrolled of which 390 (98%) completed the study. Of the 396 enrolled children, 271 (68%) children were of age between 2 years to 5 years (mean (SD) age: 3.5 (1.07) years) while 125 (32%) children were of age between 6 years to 10 years (mean (SD) age: 7 (1.2) years). The distribution of children across sexes was similar with 52% boys and 48% girls. The majority of children were Black or African (99%) (Table 1).

Of the 396 children who received the study drug, 193 (49%) children took the study drug with water, which included 141 (73%) children of age 2–5 years and 52 (26.9%) children of age 6–10 years. Mebendazole tablet was broken into half or quarters, to enable chewing with greater ease for 31 children (7.8%) overall, which included 29 (93.5%) children of age 2 to 5 years. Six children were withdrawn from the study due to noncompliance that the tablet was not chewed but it was swallowed; in 2 of these children, the tablet was dispersed in water to facilitate swallowing while in the remaining 4 the drug was not taken at all.

### 3.2. Baseline STH Infection

The baseline egg count evaluation in stool samples was conducted in 352 (89%) children to determine the baseline STH infection (Table 1). A total of 195 (55%) children had 1 or more STH infection at baseline, predominantly *T. trichiura* infection (*n* = 181 (51%)) followed by hookworm (*n* = 57 (16%)) and *A. lumbricoides* infections (*n* = 24 [7%]). Double infections were reported in 38 (11%) children for *T. trichiura* and hookworm while 13 (4%) children had *A. lumbricoides* and *T. trichiura* infection. Triple infections (*A. lumbricoides*, *T. trichiura*, and hookworm) were reported in 8 (2%) children (Table 2).

More children in the older age stratum (ages 6 to 10 years) (71%) had one or more STH infection(s) compared with younger children (age 2 to 5 years) (48%). The frequency of the double infection of *T. trichiura*, and hookworm was higher in children aged 6 to 10 years compared with children aged 2 to 5 years (16% versus 8%). The frequency of the triple infection of *A. lumbricoides*, *T. trichiura* and hookworm was also higher (4.5%) in children aged 6 to 10 years compared with children aged 2 to 5 years (1%).

### 3.3. Safety Assessments

Overall, 44 (11%) children reported at least one TEAE of which most common TEAEs (reported in ≥5 children) included diarrhea (3%), pyrexia and lymphadenopathy (2% each), and cough (1%) (Table 3). The frequency of TEAEs was similar between both of the age groups, except for diarrhea, which was reported only in children aged 2 to 5 years (10 children (4%)). Of these, 5 children had no helminth infection, while 4 children had light helminth infection and the evaluation of helminth intensity was not done for 1 child. Nine (2%) children reported at least one TEAE at 30 minutes after the dose (6 children aged 2 to 5 years and 3 children aged 6 to 10 years) compared with 10 (2.5%) children (9 children aged 2 to 5 years and 1 child aged 6–10 years) who reported at least one TEAE on day 4. The TEAEs that occurred 30 minutes after the dose included swelling in cervical/submandibular lymph nodes (*n* = 3), pyrexia and cough (*n* = 2 each), and diarrhea, jaundice, dyspnoea, and abnormal respiratory rate (*n* = 1 each). All TEAEs were mild to moderate in intensity. 

There was no specific correlation observed between presence or severity of baseline helminth infection and frequency of TEAEs, mean vital sign values and physical examinations. The percentage of children with TEAEs was similar for those with light helminth infection at baseline (23 (13%) of 181 children) and those without helminth infection at baseline (18 (11%) of 157 children) (Table 4). 

Of all the TEAEs, there was only a modest difference in the proportion of children with pyrexia in children with light helminth infection (7 (4%) out of 181 children) compared with children with no infection (4 (3%) out of 157 children) and those with moderate infection (0 out of 14 children). Of the 4 children without any infection who developed pyrexia, 2 children had pyrexia within 30 minutes after the dose while the rest of the children with or without helminth infection and had pyrexia, developed pyrexia within 1 to 3 days following mebendazole treatment. One out of 14 children with moderate helminth infection reported of dizziness which was not reported any of the other groups (Table 4). 

There were no deaths or serious TEAEs reported during this study. None of the TEAEs led to the study discontinuation.

## 4. Discussion

Zanzibar has a tropical climate, is highly densely populated, and the 1-million people living on the islands live in unsanitary conditions. All these factors sustain high prevalence and high transmission of STH infections. Pemba Island in Zanzibar, Tanzania, has a high prevalence of STH infections (approximately 70%) [13], and hence a single study center in Pemba Island that could provide a sufficient number of preschool and school-age children with STH infection was chosen for the study. In Zanzibar, the Helminth Control Program based on periodic chemotherapy in school children with mebendazole (500 mg) albendazole (400 mg), or praziquantel (40 mg/kg) is ongoing since 1995. Other interventions like Elimination of Lymphatic Filariasis based on the community yearly treatment with ivermectin plus albendazole was carried out from 2001 to 2006. As a result of these deworming interventions done earlier, the intensity of STH infections in this study was found to be mainly light; however, the finding of still relatively high prevalence especially of *T. trichiura* reflects the high reinfection and low susceptibility of this organism to the single-dose benzimidazole treatment. 

Currently, four antihelmintics that are commonly used to treat STH infections are levamisole, pyrantel, and the benzimidazole anthelmintics (mebendazole and albendazole) [14]. Of these, levamisole and pyrantel show limited effect on *T. trichiura* [15]. The safety of their administration in the public health community-based or school-based campaigns has been reviewed and their safety profile is such that these drugs can be administered by nonhealth personnel (e.g., teachers, community drug distribution) after simple training and with minimal medical supervision [5].

Mebendazole has proven efficiency and tolerability and is ideally suited for PC campaigns because of its single-dose regimen as a 500 mg tablet. Mebendazole has a robust safety profile [16]. However, the formulation used must be such that it can treat the youngest, most vulnerable, children at risk. Younger children (i.e., those <5 years old) may not be able to swallow the 500 mg tablet and the WHO recommends that these children be treated with chewable tablets in PC programs. Since only 500 mg mebendazole solid oral tablets are currently available for use in these programs, children with STH infections may go untreated, which could lead to compromised growth and development in addition to reinfection of their treated older siblings and other family and community members. In an effort to treat the younger children, sometimes tablets are crushed and administered with water; however, this requires additional work and the medicine is bitter, making this a less-than-ideal method of treating the youngest children. Liquid formulations or suspensions available for young children are not practical for use in the MDA programs due to cost concerns and storage limitations. A chewable tablet formulation, which can be crushed and administered with a small amount of water for those under 36 months, can be administered successfully to young children with a low rate of choking. Data from Madagascar's vitamin A plus deworming round in October 2006 and Rwanda's measles campaign in September 2006 reported choking of the deworming tablet in 1% and 0.1% respectively of the children aged 1 to 3 years [8]. Of note, there were no reports of choking with the 500 mg chewable mebendazole tablet in the current study.

A 500 mg chewable mebendazole tablet was, hence, developed to treat young children for STH infection in such MDA programs. In the current study, the safety profile of this chewable mebendazole tablet was demonstrated to be similar to that of the currently marketed mebendazole solid oral tablet [6]. 

Although there were no children with severe infections included in the current study, available data suggests that the safety profile of the mebendazole chewable tablet is not correlated with the type or degree of infection. The incidence of adverse events reported in the present study was low (11%) and comparable with earlier studies with albendazole [17]. The commonly reported TEAEs in this study include pyrexia, diarrhea, lymphadenopathy, and cough. These findings are similar to those reported with mebendazole [18]. Since the study did not have a placebo comparator, no determination of adverse drug reactions could be made. However, it should be noted that only one adverse event (diarrhea) was considered by the investigator to be probably related to mebendazole administration.

All 6 children (2%) who discontinued from the study did so due to noncompliance with the study drug administration (the 6 children spat out the study drug, refused to chew and swallow the drug, or had the drug suspended in water to facilitate swallowing). In addition, almost half of the children (49%) took the study drug with water of which 73% were children 2 to 5 years old. For some children, especially those aged 2 to 5 years, the mebendazole tablet was broken into pieces to enable them to chew the tablet with greater ease. Whether the noncompliance or difficulty in chewing or swallowing was a result of taste, texture, or size of the tablet is unknown. In those noncompliant younger children, great care was taken in collaboration with their caregiver (mainly mothers) to convince the child to chew and swallow the tablets. The children were never forced to chew or swallow the tablets, though, in some instances, a substantial length of time was required to complete this process. This aspect should be taken into account in campaigns when millions of children are treated and time can be a pressing need. 

This study will facilitate the production of chewable mebendazole that could be more easily administered to children in the age range of 2 to 10 years in STH endemic areas. In these children, especially the younger ones, STH infections have the potential to affect brain and cognitive development [19, 20] and this has a major impact on their ability to work and lead productive lives as adults. Thus, STH infections among children have a tremendous impact on individuals, communities, and a nation's economic productivity making these infections a major global health problem with far-reaching health and economic consequences [19]. Johnson & Johnson's annual donation commitment of mebendazole 500 mg tablets has scaled up from 50 to 200 million since 2011, and the chewable tablet, once approved, will expand the age range of treated individuals to cover children of pre-school age for health-related benefits.

## 5. Conclusions

Single-dose administration of mebendazole 500 mg chewable tablet was found to be safe and well tolerated in children between 2 and 10 years of age. This alternate formulation may be helpful in PC campaigns to treat young children who have difficulty in swallowing the currently used solid tablet.

## Figures and Tables

**Table 1:** Patient demographics and baseline characteristics.

	2–5 years	6–10 years	Total
	(*N* = 271)	(*N* = 125)	(*N* = 396)
Sex, *n* (%)			
Boys	132 (49)	74 (59)	206 (52)
Girls	139 (51)	51 (41)	190 (48)
Race, *n* (%)			
White	3 (1)	1 (1)	4 (1)
Black or African	267 (99)	124 (99)	391 (99)
Not reported	1 (0.4)	0	1 (0.3)
Age (yrs)			
Mean (SD)	3.5 (1.07)	7 (1.19)	4.6 (1.94)
Baseline BMI (kg/m^2^)			
*N*	268	125	393
Mean (SD)	14.5 (1.99)	14.5 (1.97)	14.5 (1.98)
Subgroup egg counts, *n* (%)			
Not done^a^	31 (11)	13 (10)	44 (11)
No infection^b^	124 (46)	33 (26)	157 (40)
Light^c^	109 (40)	72 (58)	181 (46)
Moderate^d^	7 (3)	7 (6)	14 (3)
*A. lumbricoides* egg count, *n* (%)			
No infection^b^	226 (83)	102 (82)	328 (83)
Light^c^	10 (4)	6 (5)	16 (4)
Moderate^d^	4 (1)	4 (3)	8 (2)
*A. lumbricoides* eggs per gram of feces^e^			
Mean (SD)	249.2 (1805.36)	399.4 (2359.70)	297.0 (1996.27)
*T. trichiura* egg count, *n* (%)			
No infection^b^	131 (48)	40 (32)	171 (43)
Light^c^	105 (39)	66 (53)	171 (43)
Moderate^d^	4 (1)	6 (5)	10 (2)
*T. trichiura* eggs per gram of feces^e^			
Mean (SD)	143.3 (355.79)	244.7 (360.54)	175.6 (359.92)
Hookworm egg count, *n* (%)			
No infection^b^	212 (78)	83 (66)	295 (74)
Light^c^	28 (10)	29 (23)	57 (14)
Hookworm eggs per gram of feces^e^			
Mean (SD)	43.0 (196.65)	99.9 (264.28)	61.1 (221.63)

BMI: body mass index.

^a^Egg count evaluation not conducted.

^b^Negative finding for egg count evaluation.

^c^Egg count evaluation finding is (*A. lumbricoides* (epg)): 1–4,999; (*T. trichiura* (epg)): 1–999; (Hookworms (epg)): 1–1,999.

^d^Moderate: egg count evaluation finding is (*A. lumbricoides* (epg)): 5,000–49,999; (*T. trichiura* (epg)): 1,000–9,999; (Hookworms (epg)): 2,000–3,999.

^e^For eggs per gram of feces, the number of children included were 2–5 years *n* = 240; 6–10 years *n* = 112; total *n* = 352.

For children infected with multiple parasites, the grouping is based on the most severe infection.

**Table 2:** Prevalence of soil-transmitted helminth infections based on the baseline egg counts evaluation.

	2–5 years old	6–10 years old	Total
	(*N* = 271)	(*N* = 125)	(*N* = 396)
Parasitic infections, *n* (%)			
*N*	240	112	352
No infection	124 (52)	33 (29)	157 (45)
* A. lumbricoides *	2 (1)	1 (1)	3 (1)
*T. trichiura *	77 (32)	45 (40)	122 (35)
Hookworm^#^	5 (2)	6 (5)	11 (3)
*A. lumbricoides* and *T. trichiura *	9 (4)	4 (4)	13 (4)
*T. trichiura* and hookworm^#^	20 (8)	18 (16)	38 (11)
*A. lumbricoides, T. trichiura,* and hookworm^#^	3 (1)	5 (4)	8 (2)

Children with no baseline egg count evaluations are not included in the table.

^#^ *Necator americanus* and *Acylostoma duodenale.*

**Table 3:** TEAEs observed in at least 1% of the patient population.

	2–5 years	6–10 years	Total
Type of TEAEs	(*N* = 271)	(*N* = 125)	(*N* = 396)
	*n* (%)	*n* (%)	*n* (%)
Total number of children with TEAE	31 (11)	13 (10)	44 (11)
Diarrhea	10 (4)	0	10 (3)
Pyrexia	8 (3)	3 (2)	11 (3)
Lymphadenopathy	5 (2)	3 (2)	8 (2)
Cough	5 (2)	0	5 (1)

TEAE: treatment-emergent adverse event.

**Table 4:** TEAEs by intensity of helminth infection.

	Not done	No infection	Light	Moderate	Total
	(*N* = 44)	(*N* = 157)	(*N* = 181)	(*N* = 14)	(*N* = 396)
	*n* (%)	*n* (%)	*n* (%)	*n* (%)	*n* (%)
Total number of children with adverse events	2 (5)	18 (11)	23 (13)	1 (7)	44 (11)
Diarrhea	1 (2)	5 (3)	4 (2)	0	10 (3)
Abdominal pain	0	1 (1)	1 (1)	0	2 (1)
Abdominal distension	0	0	1 (1)	0	1 (<1)
Aphthous stomatitis	0	1 (1)	0	0	1 (<1)
Nausea	0	1 (1)	0	0	1 (<1)
Oral pain	0	0	1 (1)	0	1 (<1)
Pyrexia	0	4 (3)	7 (4)	0	11 (3)
Lymphadenopathy	0	5 (3)	3 (2)	0	8 (2)
Cough	0	3 (2)	2 (1)	0	5 (1)
Asthma	0	0	1 (1)	0	1 (<1)
Dyspnoea	0	1 (1)	0	0	1 (<1)
Epistaxis	0	1 (1)	0	0	1 (<1)
Rhonchi	0	1 (1)	0	0	1 (<1)
Respiratory rate increased	0	0	2 (1)	0	2 (1)
Cardiac murmur	1 (2)	0	0	0	1 (<1)
Respiratory rate	0	0	1 (1)	0	1 (<1)
Bronchitis	0	2 (1)	0	0	2 (1)
Upper respiratory tract infection	0	1 (1)	0	0	1 (<1)
Urinary tract infection	0	1 (1)	0	0	1 (<1)
Rash popular	0	0	3 (2)	0	3 (1)
Dizziness	0	0	0	1 (7)	1 (<1)
Headache	0	0	1 (1)	0	1 (<1)
Jaundice	0	0	1 (1)	0	1 (<1)

TEAE: treatment-emergent adverse event.
